# Fabrication of Boron-Doped Diamond Film Electrode for Detecting Trace Lead Content in Drinking Water

**DOI:** 10.3390/ma15176013

**Published:** 2022-08-31

**Authors:** Liang Wu, Xinghong Liu, Xiang Yu, Shijue Xu, Shengxiang Zhang, Shiman Guo

**Affiliations:** School of Materials Science and Technology, China University of Geosciences (Beijing), 29 Xueyuan Road, Beijing 100083, China

**Keywords:** boron-doped diamond, electrode, water detection, heavy metal pollutant

## Abstract

This work aimed to fabricate a boron-doped diamond film electrode for detecting trace amounts of lead in drinking water so as to safeguard it for the public. Available detectors suffer from high costs and complex analytical processes, and commonly used electrodes for electrochemical detectors are subject to a short life, poor stability, and secondary pollution during usage. In this work, a boron-doped diamond (BDD) electrode was prepared on a porous titanium substrate, and the microstructure and electrochemical properties of the BDD electrode were systematically studied. Moreover, the stripping parameters were optimized to obtain a better signal response and determine the detection index. As a result, diamond particles were closely arranged on the surface of the BDD electrode with good phase quality. The electrode showed high electrochemical activity, specific surface area, and low charge transfer resistance, which can accelerate the stripping reaction process of Pb^2+^. The BDD electrode presented a low detection limit of 2.62 ppb for Pb^2+^ under an optimized parameter set with an enrichment time of 150 s and a scanning frequency of 50 Hz. The BDD electrode also has good anti-interference ability. The designed BDD electrode is expected to offer a reliable solution for the dilemma of the availability of metal electrodes and exhibits a good application prospect in the trace monitoring of Pb^2+^ content in drinking water.

## 1. Introduction

Increasing concerns about Pb^2+^ pollutants in drinking water have prompted the development of reliable analytical techniques for water quality monitoring. The rapid development of industry has caused serious water pollution and threatens the security of water drinking for over 1 billion people around the world [1]. Heavy metal pollutants are intractable pollutants that cause water quality deterioration and have extensive sources and abundant transmission channels such as industrial, agricultural, and domestic wastewater, atmospheric deposition, etc. [2,3]. Among them, Pb^2+^ is a typical stubborn heavy metal pollutant due to its characteristics of difficult degradation, easy accumulation, and high toxicity [4]. The excessive intake of Pb^2+^ can cause irreversible damage to the nervous system, internal organs, and reproductive system of the human body. The World Health Organization recommends a maximum lead content of 10 ppb in drinking water [5,6]. The detection of Pb^2+^ in drinking water is thus crucial for preventing water pollution and ensuring the safety of drinking water. Available methods for detecting trace Pb^2+^ include liquid chromatography, atomic absorption spectrometry, and flow injection analysis. The available methods suffer from high costs and complex analytical processes, and their applications for water quality monitoring are therefore constrained [7]. Instead, electrochemical analysis technologies such as square wave stripping voltammetry and differential pulse voltammetry present good potential due to their high sensitivity and fast signal response [8]. Therefore, it is of significance to develop water quality monitoring sensors based on electrochemical analyses.

The detection electrode is the core component of an electrochemical detector and determines its analytical capability. Currently, commonly used detection electrodes include mercury film electrodes and metal film electrodes, and the metal film electrodes include bismuth, antimony, gold, etc. [9]. Unfortunately, such electrodes are subject to poor stability and short life. It is thus imperative to develop new electrode materials. Being a new carbon material, BDD (boron-doped diamond) has both the good physical and chemical characteristics of diamonds and the functional performance of semiconductors. High hardness, excellent chemical stability, and good electrochemical characteristics (low background current, high potential window) make BDD applicable in the fields of supercapacitors and advanced oxidation technologies [10]. Developing heavy metal analysis electrodes based on BDD can be expected to obtain high stability and reliability in water quality monitoring. However, there is still a lack of systematic research on using BDD electrodes to detect Pb^2+^ content in drinking water.

In this work, a BDD electrode is prepared on a titanium slice to detect trace Pb^2+^ in water. The microstructural and electrochemical behavior of the prepared BDD electrodes are systematically investigated and the Pb^2+^ detection ability is also explored to evaluate the application potential of BDD electrodes in heavy metal monitoring.

## 2. Experimental Section

### 2.1. Reagents and Instruments

All reagents used were of analytical grade. The lead ion standard solution was purchased from the National Institute of Standards and Materials, and the concentration required for the solution to be tested was set by mixing it with ultrapure water. Sodium sulfate, potassium ferricyanide, and boron trioxide were purchased from Shanghai McLean Biochemical Co., Ltd. (Shanghai, China); anhydrous ethanol was provided by the Shanghai Sinopharm group; nanodiamond (10 nm) was purchased from Nanjing Hongde Material Co., Ltd. (Nanjing, China); and the titanium substrate was purchased from Shaanxi Huanya Senna Hydrogen Energy Technology Co., Ltd. (Baoji, China).

The BDD film electrode was prepared in a hot-filament chemical vapor deposition system (HFCVD, HF800, Beijing Wald Diamond Tools Co., Ltd. (Beijing, China)). The micromorphology of the electrode was observed using a scanning electron microscope (SUPRA55, Carl Zeiss AG, Oberkochen, Germany). The binding structure was detected using a Raman confocal microscope (LabRAM HR Evolution, HORIBA JobinYvon, Edison Township, NJ, USA). The electrochemical test was carried out in an electrochemical workstation (CHI 660E, Shanghai Chenhua, Shanghai, China).

### 2.2. Preparation of BDD Electrode

A titanium metal slice was used as the substrate of the BDD electrode. The cleaned substrate was placed in a diamond seed solution (5 g ND (nanodiamond)/20 mL ethanol) for 10 min for the seeding treatment.

The substrate was then dried and placed in an HFCVD chamber for deposition. Diboron trioxide was dissolved in ethanol as a doping boron source and was brought into the chamber through hydrogen gas. In the nucleation stage, methane was used as a carbon source, hydrogen was used as an etching gas, and the ratio of H_2_:CH_4_ was set as 10:1000 sccm for 0.5 h. In the growth stage, the gas flow of H_2_ (bubbling) was adjusted to C_2_H_5_OH + H_2_ + B_2_O_3_:H_2_ = 25:50:1000 sccm for 7.5 h. The layer thickness could be determined by the product of the deposition rate and deposition time. The main deposition parameters included the C/H ratio (2.4%), B/C ratio (6000 ppm), deposition pressure (3 KPa), and deposition temperature (700 °C).

### 2.3. Electrochemical Measurement

BDD film was used as the working electrode (10 × 10 mm^2^), a saturated glyceryl electrode was used as the reference electrode (3 M KCl), and a platinum sheet was used as the counter electrode (10 × 15 mm^2^). A cyclic Voltammetry (CV) test was used to investigate the electrode potential window, background current, and reaction kinetics in the electrolytes of a 0.1 M Na_2_SO_4_ solution and a 0.1 M K_3_[Fe(CN)_6_] solution. Electrochemical impedance spectroscopy (EIS) was used to detect the electrochemical reaction process in a frequency range of 10^−2^–10^5^ Hz. Square wave dissolution voltammetry (SWASV) was used to assess Pb^2+^ in the water under the conditions of an amplitude of 20 mV, a step potential of 4 mV, and a deposition potential of −0.8 V. The limit of detection (LoD) of the BDD electrode was calculated using the formula 3N/S, where N is the electrode noise value and S is the electrode sensitivity.

## 3. Results and Discussion

### 3.1. Structural Characterization of BDD Electrode

**Figure 1:**
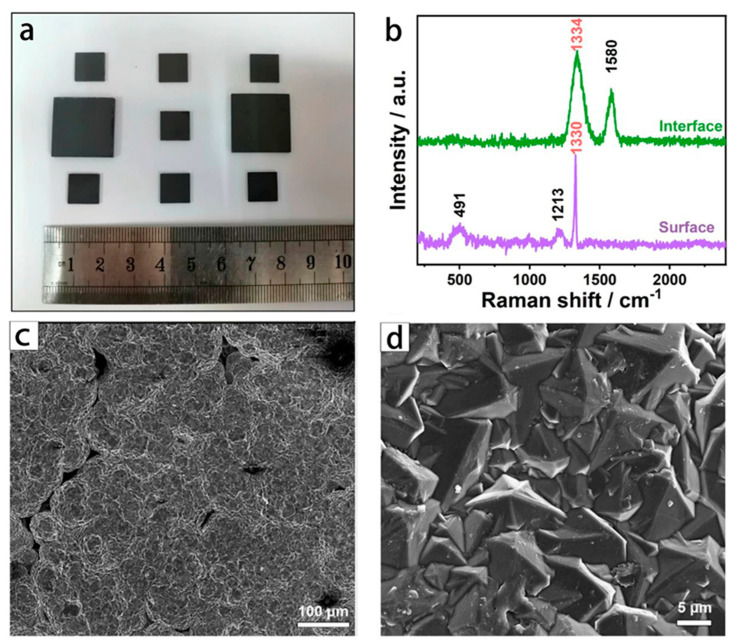
Structure of BDD electrode. (**a**) Digital photographs; (**b**) Raman spectra; (**c**,**d**) SEM images at two magnifications.

Figure 1a shows a physical view of the BDD electrode prepared in this work. As shown in Figure 1a, the electrode surface was smooth, and there were no macroscopic defects such as flaking and cracking, suggesting a good adhesion from the BDD film to the substrate. Also, Raman scattering spectra were used to characterize the binding structures of the BDD film. Figure 1b shows the Raman spectra of the prepared BDD electrode at both the surface and interface. In Figure 1b, it can be seen that the diamond phase has a good quality at the BDD surface and boron doping is achieved. For the interface of the BDD electrode, two characteristic peaks can be observed at 1334 cm^−1^ and 1580 cm^−1^, corresponding to the D (Diamond) band (*sp^3^*-C) and G (graphite) band (*sp^2^*-C), respectively [11]. The D band is the first-order Raman characteristic peak of the diamond phase and can be used as direct evidence to determine the diamond formation. The G band is attributed to the impurity phase similar to amorphous carbon in the BDD. The significant G band at the interface indicates a poor phase formation at the initial growth stage of the BDD film due to the presence of a large amount of overlapped amorphous carbon.

On the BDD electrode surface, the absence of a G peak at 1580 cm^−1^ indicates an improvement in the diamond phase formation. In addition, the appearance of a new characteristic peak at 491 cm^−1^ can be attributed to phonon scattering brought about by lattice changes after doping boron [12]. Meanwhile, the D peak at the BDD surface has “blue-shifted” appearing at 1330 cm^−1^ compared with that at the interface. This phenomenon is due to the “Fano” effect caused by boron doping [13]. These two phenomena jointly confirm that boron was successfully doped into the diamond lattice to form the BDD film. The BDD film was mainly composed of the diamond phase and boron was doped. This is favorable for reducing the electrode noise and widening the potential window. The amorphous carbon phase at the interface can be used as a conductive medium to facilitate the transmission of electrical signals. Such a structure of the BDD electrode can be expected to achieve good electrochemical behavior and enhanced detection performance.

Subsequently, the microscopic morphology of the BDD electrode was observed using SEM. Figure 1c,d show the SEM images of the electrode at two magnifications. As shown in Figure 1c, the porous structure of the substrate was preserved after the deposition of the BDD film. As shown in Figure 1d, the average size of the diamond grain was about 8 μm and the grain morphology was a regular tetrahedral structure. The tetrahedral structure is a typical morphological feature of a diamond grain with a (111) crystal plane and this indicates that the film has a preferred orientation along the (111) crystal plane [14]. The (111) crystal plane is the easily exposed surface of the B element, and the preferred orientation is beneficial for increasing the active sites of the electrode and enhancing heavy metal detection. In short, favorable microscopic morphology, good phase structure, and preferred orientation of the film jointly facilitate a good electrochemical analysis capability.

### 3.2. Electrochemical Characterization of BDD Electrode

**Figure 2:**
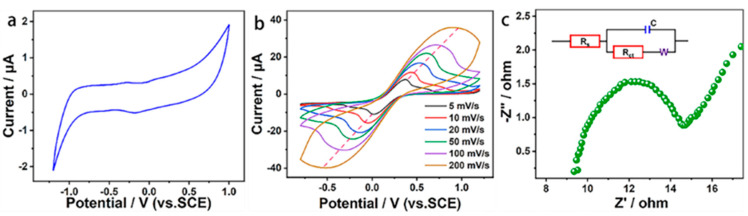
Electrochemical behavior of BDD electrode. (**a**) Cyclic voltammetry characteristic curve of BDD electrode in 0.1 M Na_2_SO_4_ solution at scanning speed of 10 mV/S; (**b**) Cyclic voltammetric curves of BDD electrode in 0.1 M K_3_[Fe(CN)_6_] electrolyte at different scanning speeds; (**c**) Nyquist diagram of BDD electrode in 0.1 M potassium ferricyanide solution.

Good electrochemical behavior is a guarantee of efficient heavy metal detection of a BDD electrode. Firstly, the potential window and background current of the BDD electrode were measured by cyclic voltammetry, as shown in Figure 2a. As can be seen in Figure 2a, the BDD electrode had no hydrogen evolution and oxygen absorption reactions in the potential range of −1.2 V to +1.0 V and the electrode potential window was as high as 2.2 V. This wide potential window is attributed to the weak adsorption of *sp^3^*-C on the BDD electrode to the reaction intermediates in the solution. The potential window of 2.2 V can fully ensure the dissolution analysis of Pb^2+^ by the BDD electrode. In addition, the electrode also showed a very low background current, which can suppress the electrode noise and improve the signal ratio and sensitivity of the electrode. On the other hand, the low background current of the electrode represents the low electric double layer capacitance, indicating that there are fewer charge sites on the electrode surface, which may hinder the charge transfer on the electrode surface [14].

Subsequently, the electrochemical reaction kinetics of the BDD electrode were investigated using potassium ferricyanide as the redox pair. Figure 2b shows the cyclic voltammetric characteristics of the BDD electrode at different scan rates. In Figure 2b, obvious oxidation and reduction peaks can be observed at all six scan rates, indicating a good electrochemical capability. The intensity of oxidation and reduction peaks increases with an increase in the scan rate. A linear fit between the rate’s square root and the peak current values reveals a good linear relationship. This means that the diffusion process dominates the electrochemical reactions on the BDD electrode, and the electrochemical reactions occurring on the electrode are quasi-reversible processes [15]. Moreover, the active surface area reaches 4.38 cm^2^, calculated using the Randles–Sevcik equation [16]. The above results confirm that the BDD electrode had an active surface and facilitated a favorable electrochemical behavior.

Furthermore, the charge transfer capability of the electrode was evaluated by an impedance test. Figure 2c shows the Nyquist plot of the poles in the potassium ferricyanide electrolyte. In Figure 2c, a semicircular shape occurs in the high-frequency range. This is the embodiment of the charge transfer process and corresponds to the charge transfer resistance labeled as Rct in the equivalent circuit diagram. The calculated value of Rct was 6.54 Ω. Such a value indicates that the electrode has a low charge transfer resistance and can ensure the smooth progress of the electrochemical reaction. The Nyquist plot shows a linear shape in the low-frequency region. This is the embodiment of the diffusion process and is involved with the Warburg impedance in the equivalent circuit diagram. The linear angle is about 45°, indicating a stable diffusion process. In short, the BDD electrodes had a lower charge transfer resistance and were beneficial for improving the signal response of the electrode.

### 3.3. Determination of Trace Pb^2+^ in Water

#### 3.3.1. Dissolution Parameter Optimization

**Figure 3:**
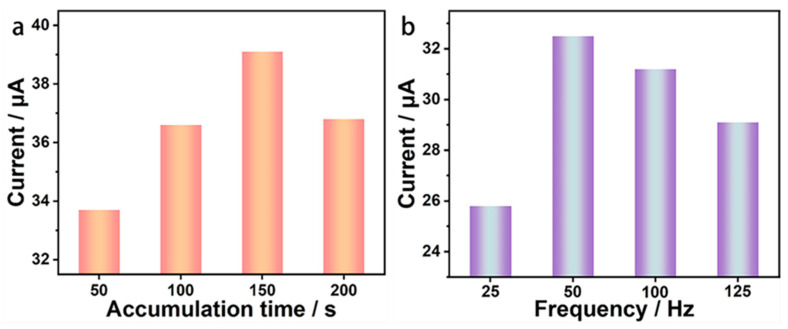
Effect of enrichment time and scan frequency on the signal response of the BDD electrode. (**a**) Dissolved peak current values of BDD electrode for Pb^2+^ at four enrichment times; (**b**) Dissolved peak current values of BDD electrode for Pb^2+^ at four scanning frequencies.

In order to obtain a better signal response for the BDD electrode, two specially selected experimental parameters enrichment time and scanning frequency were optimized in turn at a Pb^2+^ concentration of 30 ppb. As an important factor affecting the dissolution signal, the enrichment time is directly involved with the redox process of Pb^2+^. Figure 3a shows the dissolution peak current values at four enrichment times. In Figure 3a, the signal response shows a trend of enhancement followed by decay with the increase in the enrichment time. The electrode exhibits the lowest signal response at an enrichment time of 50 s. This enrichment time is too short to drive a large amount of Pb^2+^ to participate in the dissolution reaction. The extension of the enrichment time enhances the signal response. Consequently, the electrode achieves the highest value of the dissolution peak current at an enrichment time of 150 s and the electrode reaches the highest signal response. The reason may be that more and more heavy metal ions to be measured were reduced on the electrode surface with the extension of the enrichment time and the current signal intensity gradually increased and reached the maximum value [17]. After that, the electrode signal response deteriorated as the current value of the dissolved peak decayed slightly when the enrichment time was extended to 200 s. This could have been due to the impurities in the solution system. The impurity ions in the solution system gradually participated in the dissolution process, interfering with the reduction in the ions to be measured on the electrode and suppressing the current value. Thus, 150 s was selected as the preferred enrichment time due to its highest signal response.

Scanning frequency is another important factor affecting the dissolution signal and the scanning frequency affects the signal response of the ion to be measured by influencing the input process of the voltage signal. To this end, the peak current values of Pb^2+^ dissolved at four scanning frequencies (25 Hz, 50 Hz, 100 Hz, 125 Hz) were examined sequentially at an enrichment time of 150 s. Figure 3b shows the dissolution peak current values at the four scanning frequencies. In Figure 3b, it can be seen that the signal response exhibits a similar trend of enhancement followed by decay with the increase in the scanning frequency, and the maximum value is obtained at 50 Hz. A low scan frequency leads to a poor signal response (25 Hz). In this case, the interaction efficiency between the input voltage and the ions to be measured was low, and it was difficult to oxidize the reduced heavy metal ions by the reverse scan voltage, resulting in low dissolved peak current values. The signal response enhanced with the increase in the scanning frequency and reached the optimum at 50 Hz. The scanning frequency at this point could guarantee the full reduction and oxidation of the ions to be measured and the peak current reached the maximum value. Subsequently, the signal response slightly decreased with a further increase in the scanning frequency to 100 and 125 Hz. This could have been due to the fact that the excessive scanning frequency caused residual reduced metal substances on the electrode surface, which suppressed the dissolved peak current value.

In this way, an enrichment time of 150 s and a scanning frequency of 50 Hz were selected as the optimized dissolution parameters for the following experiments.

#### 3.3.2. Sensitivity Detection

**Figure 4:**
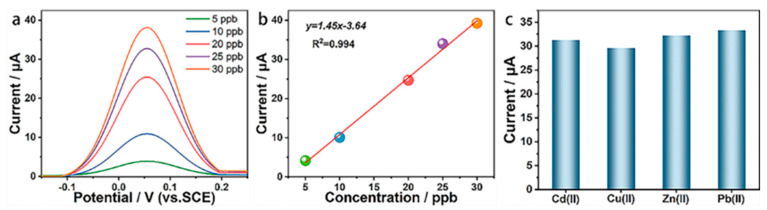
Detection performance of BDD for Pb^2+^. (**a**) Dissolution curves of five Pb^2+^ concentrations; (**b**) Linear fitting curve between Pb^2+^ concentration and dissolution peak current; (**c**) Dissolution peak current before and after addition of interfering ions.

The detection ability of electrodes is a unique performance indicator for evaluating their adaptability to real water analysis. Drawing a standard curve is a reliable method for obtaining the electrode sensitivity, detection limit, and linear range. The optimized dissolution parameter combination was thus used to detect the concentration of Pb^2+^ in the solution with the gradient change, where the dissolution peak current value and Pb^2+^ concentration were linearly fitted. Figure 4a shows the dissolution curves of the BDD electrode for the five Pb^2+^ concentrations. In Figure 4a, the dissolution peak current value gradually increases with the increase in the concentration and the dissolution peak potential value has no obvious drift. Figure 4b shows the linear fitting curve between the Pb^2+^ concentration and its dissolution peak current value. As shown in Figure 4b, the concentration of Pb^2+^ maintains a linear relationship with the dissolution peak current in the range of 5–30 ppb, and the linear correlation (R^2^) of the fitting curve is as high as 0.994. The standard curve equation is y = 1.45x − 3.64 and the electrode sensitivity is 1.45 μA L μg^−1^ cm^−2^, reflected by the slope of the standard curve. The electrode LoD (3N/S) is further calculated as 2.62 ppb. The above results indicate that the prepared BDD electrode can realize the sensitive detection of trace Pb^2+^ in water.

A comparative analysis was conducted to evaluate the detection performance of the fabricated BDD electrodes with that of the relevant electrodes reported in the literatures, as listed in Table 1. It can be seen in Table 1 that the BDD electrode exhibits a superior LoD in a wider linear range compared with the other electrodes in the literatures. Consequently, this comparative analysis demonstrates that the BDD electrode may be applicable for determining trace Pb^2+^ in actual water.

#### 3.3.3. Anti-Interference Ability

The complex components of the actual body of water are a hindrance to the BDD electrode for its practical application, and good anti-interference ability can guarantee the electrode will conduct highly selective analyses to cope with the complex detection environment. Cd^2+^, Cu^2+,^ and Zn^2+^ were added to the solution system to be measured for electrochemical analysis, and the anti-interference ability was evaluated by recording the changes in the peak current values of the dissolved Pb^2+^. Cd^2+^, Cu^2+,^ and Zn^2+^ were added at the same concentration as Pb^2+^, all at 30 ppb. Figure 4c shows the dissolved peak current values of the pure Pb^2+^ solution and the mixed solutions with the addition of Cd^2+^, Cu^2+,^ and Zn^2+^. As shown in Figure 4c, the pure Pb^2+^ solution has the highest signal intensity, and the dissolution signal of Pb^2+^ does not change significantly after the sequential addition of Cd^2+^, Cu^2+^, and Zn^2+^, and only a slight attenuation occurs. This result clearly illustrates the good anti-interference ability of the BDD electrode and provides direct evidence for its application potential in real water bodies of a wide range.

Complex components can affect the detection performance of a BDD electrode in actual drinking water. Many researchers have made numerous efforts to explore the effects of various components and their interactions in recent decades. The components can be divided into three types, i.e., ions (heavy metals, transition metals, and counter ions); inorganic components; and organic components. As for the ions, the impact of heavy metal ions plays a leading role in the anti-interference ability and is described above. Apart from transition metals, water can also contain corresponding counter ions such as sulfate ions, sulfite ions, chloride ions, nitrate ions, nitrite ions, hydroxyl groups, etc. [23]. The inorganic components include carbon monoxide and carbon dioxide, oxygen, industrial dust, etc. [24]. The organic components include amines, mercaptans, phenols, arenes, halogen-containing organic substances, etc. [25]. The presence of the components can affect the detection process and results and their effects can vary from case to case. Consequently, the complexity of the components, their interactions, and the uncertainty of unknown structures are related to the electrode’s applications.

## 4. Conclusions

In this work, a BDD film electrode was developed to detect trace amounts of Pb^2+^ in water. Based on a systematical investigation of the microstructural and electrochemical performance, the dissolution parameters of the BDD electrode were optimized, and heavy metal detection of the electrode was also evaluated. The main conclusions are (1) the diamond grains were arranged and submerged on the BDD electrode surface and featured a high phase quality; (2) the electrode had a wide potential window (2.2 V), a low background current, and a large electrochemical active area (4.38 cm^2^), and the low charge transfer resistance (Rct = 6.54 ω) could promote the dissolution reaction process of Pb^2+^; and (3) the electrode showed excellent selectivity for Pb^2+^ of 1.45 μA L μg^−1^ cm^−2^ in a linear range of 5–30 ppb and a low detection limit of 2.62 ppb. The BDD electrode developed in this work is expected to achieve the stable and reliable monitoring of Pb^2+^ content in drinking water.

## Figures and Tables

**Table 1:** Performance comparison of the BDD electrode with the other electrodes.

Electrode	Heavy Mental	LoD	Ref.
BiF_4_-CPE	Cd^2+^, Pb^2+^	54 and 93 ppb	[18]
CNTsNafion-/CPE	Cd^2+^, Pb^2+^	19.6 and 11.9 ppb	[19]
HgSPE	Pb^2+^	8.9 ppb	[20]
BiRDE	Cd^2+^, Pb^2+^	5.64 and 5.85 ppb	[21]
pBDD	Pb^2+^	3.6 ppb	[22]
BDD	Pb^2+^	2.62 ppb	This work

## Data Availability

Data is contained within the article.
